# Emergency laparoscopy in underserved settings: optimizing diagnosis and management of appendicitis with ruptured hemorrhagic ovarian cyst

**DOI:** 10.1097/MS9.0000000000001736

**Published:** 2024-01-18

**Authors:** Sary Abdallah, Jana Tarchichi, Hussein Z. Alabidin Ammasri, Alaa Tarchichi, Graziella Moufawad

**Affiliations:** a Beirut Arab University School of Medicine; b Obstetrics and Gynecology Department, Beirut Arab University; c Department of Obstetrics and Gynecology, School of Medicine, Lebanese American University; d Balamand University, School of Medicine, Beirut, Lebanon; e Kharkiv National University, School of Medicine, Kharkiv, Kharkiv, Ukraine

**Keywords:** acute abdominal pain, case report, diagnostic dilemma, laparoscopy, underserved area, young female patient

## Abstract

**Introduction and importance:**

Laparoscopy is an established widely available technique for diagnosis and management. However, due to its high maintenance and expensive use, it is not readily available in emergency settings especially in underserved area hospitals. The coincidence of gynecologic and nongynecologic surgical emergencies incurs a diagnostic dilemma especially in women of reproductive age presenting with acute abdominal pain.

**Case presentation:**

This article is a case report about a woman presenting with acute abdominal pain in an underserved area and diagnosed as appendicitis.

**Clinical discussion:**

Emergency laparoscopy is so rare in underserved areas due to its high maintenance costs as well as the lack of availability of well-trained surgeons and personnel. The exceptional availability of emergency laparoscopy in her case has allowed the codiagnosis of a ruptured hemorrhagic ovarian cyst with the optimal surgical management preventing the complications that could have occurred from misdiagnosing the coincident ruptured hemorrhagic cyst.

**Conclusion:**

Emergency laparoscopy is not always available in such clinical settings and has, in our case, optimized the management and prevented an undiagnosed ruptured hemorrhagic cyst together with its complications.

## Introduction

Highlights

- Laparoscopy has an undeniable role in underserved areas to prevent morbidity and mortality.
- Disease processes particular to women should be evaluated and eliminated in the initial diagnosis of women with acute abdominal pain.
- Emergency laparoscopy if of great importance especially in cases of diagnostic dilemma.

Acute abdominal pain is a common cause of presentation to the emergency department, and it is estimated that it is responsible to 5–10% of the complaints^1^. Compared to men, women of child-bearing age presenting for this complaint pose a greater diagnostic dilemma^1^. Usually patients with acute abdominal pain are referred to a general surgeon for primary evaluation; however, in the case of women of child-bearing age an extended differential diagnosis should be kept in mind^2^. Laparoscopy is usually a well-established technique for primary evaluation or diagnosis in cases of acute abdominal pain^3^. However, in certain underserved areas in Lebanon, especially with the worsening economic crisis, emergency laparoscopy is not always available due to its high maintenance costs and the absence of well-trained surgeons able to perform such procedures. Since the 2019 economic crisis in Lebanon, an unprecedented exodus of doctors has been encountered searching for safety and stability^4^. This has mainly affected underserved area hospitals in Lebanon, which could not retain the doctors nor maintain advanced techniques to maintain the best health services. The aim of this case is to highlight the importance and the role of emergency laparoscopy and the availability of well-trained personnel in underserved areas in Lebanon for a better and adequate health-care management.

## Case presentation

### Patient history

This is the case of a 32-year-old Lebanese woman, gravida 2, para 2, aborta 0, who presented to the emergency department with a 1-day history of acute onset severe right abdominal pain associated with nausea. She describes her pain as periumbilical abdominal pain with radiation to the right lower quadrant. This patient has no past medical or surgical history. Her obstetrical and gynecological history includes two normal vaginal deliveries at term. No previous history of ovarian cysts, or ovarian torsion. She reported two episodes of vomiting and chills.

### Clinical examination

Upon presentation, vital signs were stable: blood pressure 110/63 mmHg, heart rate 87 bpm, oxygen saturation 98% on room air, and temperature 37.8°C. Upon clinical examination, a positive McBurney’s sign was noted. No palpable abdominal or pelvic masses were found.

Blood results showed slightly elevated CRP with a normal white blood cell count, and negative hcG results.

### Diagnostic imaging

**Figure 1:**
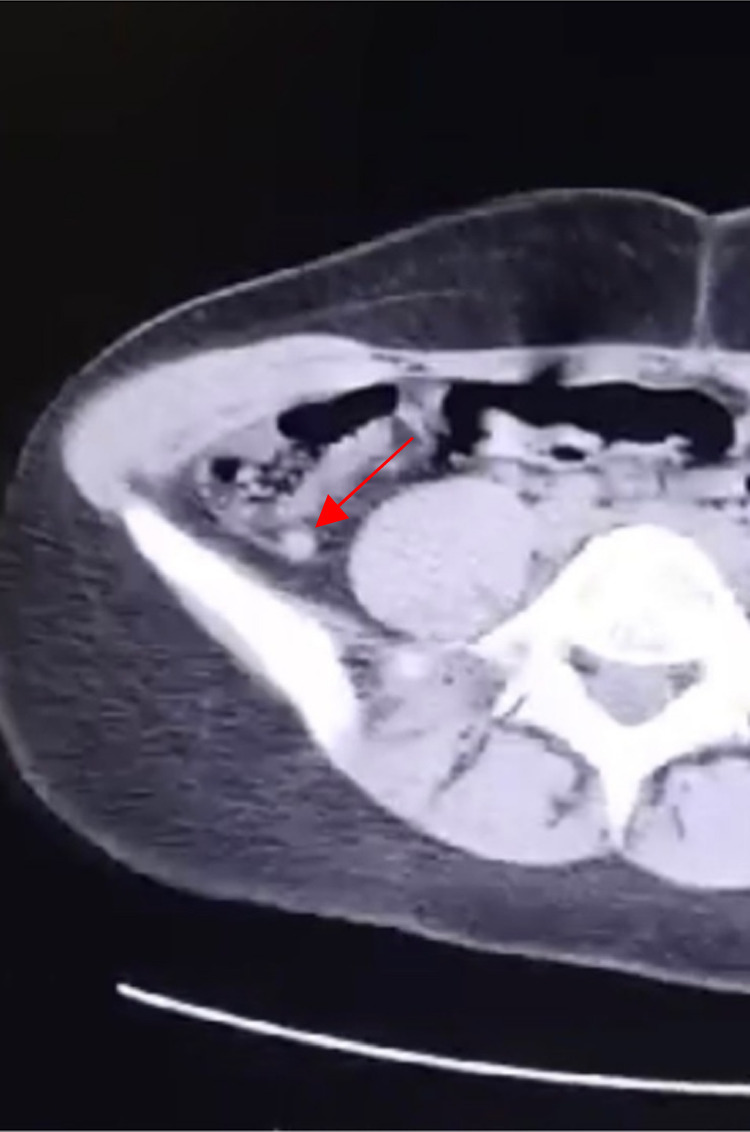
Computed tomography scan showing early signs of appendicular inflammation, arrow above.

An emergency abdomino-pelvic computed tomography scan (Fig. 1) with intravenous contrast revealed early acute appendicitis, with normal findings otherwise.

### Differential diagnoses

Both the surgeon and the radiologist were biased by an appendicular clinical picture.

### Management plan and postoperative course

**Figure 2:**
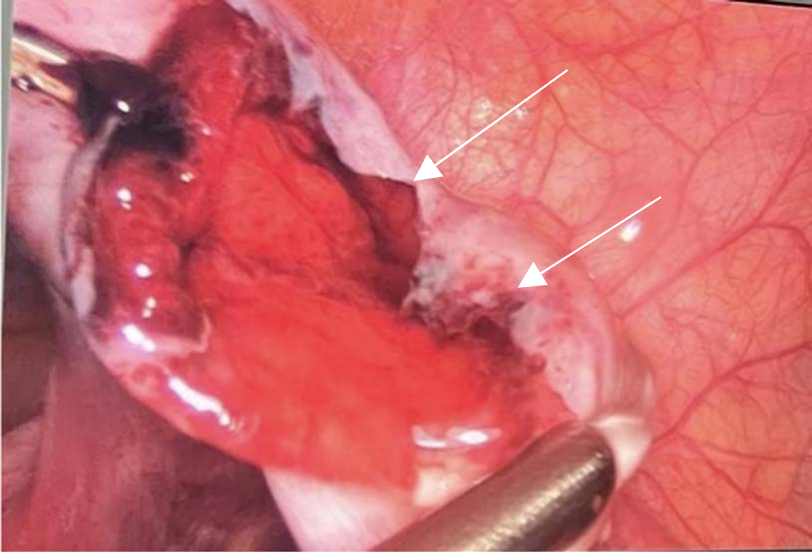
Ruptured right hemorrhagic ovarian cyst. Ovarian cyst walls depicted by fleshes above.

**Figure 3:**
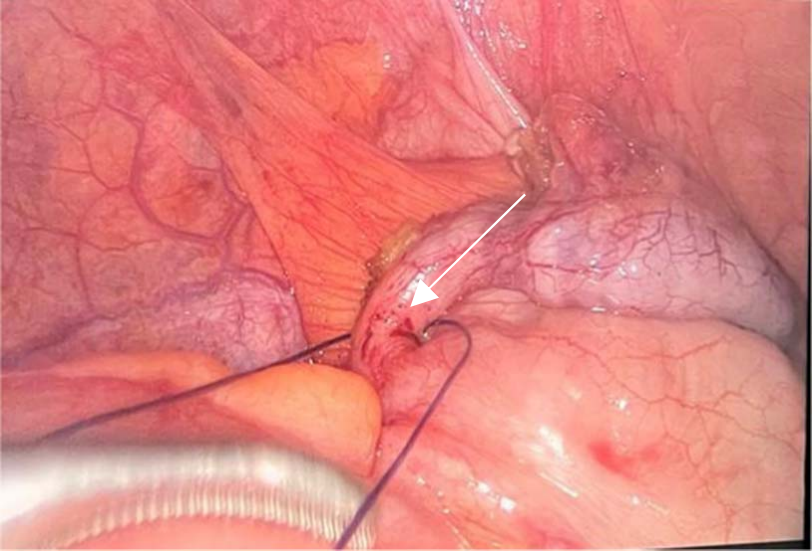
Appendectomy performed, final suturing stages in this figure.

The patient was taken to the operating room for an emergency laparoscopy performed by the general surgeon on call within a delay of 4 h from presentation. Abdominal cavity exploration revealed hemoperitoneum in the Douglas pouch. Intraoperative findings revealed appendicular early inflammation with an early ruptured right ovarian hemorrhagic cyst (Fig. 2). An appendectomy was performed (Fig. 3) together with a right ovarian cystectomy. Hemostasis was secured and the patient had a smooth intraoperative course. The specimens removed were sent to pathology.

Postoperatively, the patient had a smooth course, passed flatus on day 1 and was discharged home afterward on analgesics.

Pathology results revealed early acute appendicitis and a benign ovarian cyst.

We obtained a written informed consent from the patient for publication of the case.

The work has been reported in line with the Surgical CAse REport (SCARE) criteria^5^.

## Discussion

### Diagnostic challenges in underserved areas

This is a case reported from an underserved hospital in Lebanon where an emergency laparoscopy is not always available. Such cases are usually managed by an emergency minilaparotomy for appendectomy due to the unavailability of emergency laparoscopy in such clinical settings, whereby pelvic hemoperitoneum could have been missed together with the diagnosis of an associated ruptured ovarian cyst. The use of laparoscopy in our case has ameliorated our diagnosis, optimal surgical management, and prevented the complications of an undiagnosed ruptured ovarian cyst: hemorrhagic shock with its associated complications and poor patient outcome.

The availability of emergency laparoscopy is rare in underserved area hospitals in Lebanon. With the emerging economic crisis in Lebanon since 2019, a large number of doctors has been emigrating in search for stability and safety in other countries. Well-trained doctors are fleeing the economic instability with its associated declining income, reduced quality of care, stressful work environment, and the insecurity from deteriorating socio-political situation^4^. To add to this, the maintenance of laparoscopy needs a certain budget, which certain hospitals, especially in underserved areas fail to ensure. This case clearly depicts the importance of laparoscopy, especially in underserved hospitals, in diagnosing the coincidence of acute appendicitis and ruptured ovarian cyst which was not diagnosed clinically or via imaging techniques.

Right iliac fossa pain remains an important cause of surgical emergency department consults^3^. In particular, abdominal pain in women adds to the diagnostic dilemma. In young female patients, several differential diagnoses have to be eliminated: acute appendicitis, ectopic pregnancy, ovarian torsion, rupture hemorrhagic cyst, and others. However, misdiagnosis can occur due to several factors such as available diagnostic tools, clinical experience, patient factors^6^. In rare instances, appendicitis can occur simultaneously with a gynecological cause such as/ ruptures ovarian cyst or torsion, which can further increase the diagnostic dilemma and the management plan^2^.

Several gynecologic-related conditions can present with acute right iliac fossa pain, rendering the diagnosis of acute appendicitis in female patients unclear. Similarly, pelvic pathology can sometimes be misinterpreted as an acute intra-abdominal disease process such as diverticulitis, peritonitis, or cholecystitis^7^.

### Role of laparoscopy in optimizing diagnosis

Laparoscopy is an established widely available technique for diagnosis and management whereby a surgeon uses a camera and several trocar ports to explore the abdomino-pelvic cavity^3^. However, due to its high maintenance and expensive use, it is not readily available in emergency settings especially in underserved area hospitals. Reaching 100% accuracy in diagnosing acute Right iliac fossa pain is still unattainable despite wide advancement in laboratory and imaging techniques^8^. Thus, given that well-trained surgeons and laparoscopic techniques are available, diagnostic laparoscopy is a safe approach in the management of acute onset right iliac fossa pain^9^.

Finally, female patients frequently present with acute abdominal pain. Despite advances in laboratory and imaging techniques, the use of laparoscopy has proven to be of great importance especially in cases of diagnostic dilemma. The use of emergency laparoscopy is of utmost importance when a trained surgeon is available, and can help diagnose an undiagnosed concomitant gynecological disease process as well help in early management and prevention of associated complications and poor patient outcome.

## Conclusion

In conclusion, the availability of emergency laparoscopy in the underserved area hospital in our case has helped diagnose the coincidence of appendicitis with a ruptured ovarian hemorrhagic cyst. This has optimized adequate management and has helped prevent associated complications of misdiagnosis.

Laparoscopy remains an important diagnostic and management tool especially in underserved areas where emergency laparoscopy is not always available. The use of emergency laparoscopy can prevent severe complications and associated morbidity and mortality.

Disease processes particular to women should be evaluated and eliminated in the initial diagnosis of women with acute abdominal pain. Although rare, the concomitant occurrence of surgical and gynecological disease can be the cause of abdominal pain.

Physicians are one of the main pillars of human resources in underserved areas. Retaining these physicians in order to work and serve in such hospitals necessitates a comprehensive approach from the ministry of health and hospitals developing and implementing human resource retention strategies. International programs and sponsorships can also help ensure the maintenance of laparoscopic and advanced surgical tools for the sake of improved medical management.

Does the availability of surgical tools as well as well-trained surgeons in underserved hospitals improve the diagnosis, change the management techniques and thus improve the overall prognosis in acute settings? A question definitely worth raising in an attempt to try to implement equality and justice in medicine and surgical practices.

## Ethical approval

This paper was exempt from an institutional review board approval because it is a case report.

## Consent

Written informed consent was obtained from the patient for publication of this case report and accompanying images. A copy of the written consent is available for review by the Editor-in-Chief of this journal upon request.

## Sources of funding

Not available.

## Author contribution

S.A.: study concept and design and data interpretation; J.T.: writing the paper, review, and editing; H.Z.A.A.: data collection, review, and editing; A.T.: writing the paper and data collection; G.M.: writing the paper and study concept and design.

## Conflicts of interest disclosure

There are no conflicts of interest.

## Research registration unique identification number (UIN)

Not applicable.

## Guarantor

Sary Abdallah.

## Data availability statement

Not applicable.

## Provenance and peer review

Not commissioned, externally peer-reviewed.

## References

[1] Abdelhadi MSA. Acute abdominal pain in women of childbearing age remains a diagnostic dilemma. J Family Community Med 2001;8:45–50. PMC3437059 23008643

[2] Louis MA Doubleday AR Lin E. Abdominal pain in the female patient: a case of concurrent acute appendicitis and ruptured endometrioma. Case Rep Surg 2016;2016:2156148. 28097032 10.1155/2016/2156148 PMC5206420

[3] Afzal B Changazi SH Hyidar Z. Role of laparoscopy in diagnosing and treating acute nonspecific abdominal pain. Cureus 2021;13:e18741. 34796051 10.7759/cureus.18741 PMC8589343

[4] Nemr E Moussallem M Nemr R. Exodus of Lebanese doctors in times of crisis: a qualitative study. Front Health Serv 2023;3:1240052. 38028945 10.3389/frhs.2023.1240052 PMC10643131

[5] Sohrabi C Mathew G Maria N. The SCARE 2023 guideline: updating consensus Surgical CAse REport (SCARE) guidelines. Int J Surg Lond Engl 2023;109:1136. 10.1097/JS9.0000000000000373 PMC10389401 37013953

[6] Morino M Pellegrino L Castagna E. Acute nonspecific abdominal pain: a randomized, controlled trial comparing early laparoscopy versus clinical observation. Ann Surg 2006;244:881–888. 17122613 10.1097/01.sla.0000246886.80424.ad PMC1856631

[7] Boyd CA Riall TS. Unexpected gynecological findings during abdominal surgery. Curr Probl Surg 2012;49:195–251. 22424211 10.1067/j.cpsurg.2011.12.002 PMC3313456

[8] Andersson RE Hugander AP Ghazi SH. Why does the clinical diagnosis fail in suspected appendicitis? Eur J Surg 2000;166:796–802. 11071167 10.1080/110241500447434

[9] Rafique U Elfeky MA Bhatti K. Does diagnostic laparoscopy still have a role in the evaluation of right iliac fossa pain versus imaging techniques or experience? Cureus 2022;14:e30678. 36439602 10.7759/cureus.30678 PMC9689834

